# *Ex vivo* explant model of adenoma and colorectal cancer to explore mechanisms of action and patient response to cancer prevention therapies

**DOI:** 10.1093/mutage/geac020

**Published:** 2022-11-25

**Authors:** Sam Khan, Gareth J Miles, Constantinos Demetriou, Zahirah Sidat, Nalini Foreman, Kevin West, Ankur Karmokar, Lynne Howells, Catrin Pritchard, Anne L Thomas, Karen Brown

**Affiliations:** Leicester Cancer Research Centre, Robert Kilpatrick Clinical Sciences Building, University of Leicester, Leicester LE2 7LX, United Kingdom; Leicester Cancer Research Centre, Robert Kilpatrick Clinical Sciences Building, University of Leicester, Leicester LE2 7LX, United Kingdom; Leicester Cancer Research Centre, Robert Kilpatrick Clinical Sciences Building, University of Leicester, Leicester LE2 7LX, United Kingdom; Hope Clinical Trials Facility, Leicester Royal Infirmary, Leicester LE1 5WW, United Kingdom; Leicester Cancer Research Centre, Robert Kilpatrick Clinical Sciences Building, University of Leicester, Leicester LE2 7LX, United Kingdom; Leicester Cancer Research Centre, Robert Kilpatrick Clinical Sciences Building, University of Leicester, Leicester LE2 7LX, United Kingdom; Leicester Cancer Research Centre, Robert Kilpatrick Clinical Sciences Building, University of Leicester, Leicester LE2 7LX, United Kingdom; Leicester Cancer Research Centre, Robert Kilpatrick Clinical Sciences Building, University of Leicester, Leicester LE2 7LX, United Kingdom; Leicester Cancer Research Centre, Robert Kilpatrick Clinical Sciences Building, University of Leicester, Leicester LE2 7LX, United Kingdom; Leicester Cancer Research Centre, Robert Kilpatrick Clinical Sciences Building, University of Leicester, Leicester LE2 7LX, United Kingdom; Leicester Cancer Research Centre, Robert Kilpatrick Clinical Sciences Building, University of Leicester, Leicester LE2 7LX, United Kingdom

**Keywords:** colorectal cancer, immune modulation, patient-derived explants (PDEs), adenoma, curcumin

## Abstract

Colorectal cancer (CRC) is the second leading cause of cancer death in the UK. Novel therapeutic prevention strategies to inhibit the development and progression of CRC would be invaluable. Potential contenders include low toxicity agents such as dietary-derived agents or repurposed drugs. However, *in vitro* and *in vivo* models used in drug development often do not take into account the heterogeneity of tumours or the tumour microenvironment. This limits translation to a clinical setting. Our objectives were to develop an *ex vivo* method utilizing CRC and adenoma patient-derived explants (PDEs) which facilitates screening of drugs, assessment of toxicity, and efficacy. Our aims were to use a multiplexed immunofluorescence approach to demonstrate the viability of colorectal tissue PDEs, and the ability to assess immune cell composition and interactions. Using clinically achievable concentrations of curcumin, we show a correlation between curcumin-induced tumour and stromal apoptosis (*P* < .001) in adenomas and cancers; higher stromal content is associated with poorer outcomes. B cell (CD20^+ve^) and T cell (CD3^+ve^) density of immune cells within tumour regions in control samples correlated with curcumin-induced tumour apoptosis (*P* < .001 and *P* < .05, respectively), suggesting curcumin-induced apoptosis is potentially predicted by baseline measures of immune cells. A decrease in distance between T cells (CD3^+ve^) and cytokeratin^+ve^ cells was observed, indicating movement of T cells (CD3^+ve^) towards the tumour margin (*P* < .001); this change is consistent with an immune environment associated with improved outcomes. Concurrently, an increase in distance between T cells (CD3^+ve^) and B cells (CD20^+ve^) was detected following curcumin treatment (*P* < .001), which may result in a less immunosuppressive tumour milieu. The colorectal tissue PDE model offers significant potential for simultaneously assessing multiple biomarkers in response to drug exposure allowing a greater understanding of mechanisms of action and efficacy in relevant target tissues, that maintain both their structural integrity and immune cell compartments.

## Introduction

Colorectal cancer (CRC) is the fourth most common cancer and the second leading cause of cancer death in the UK [1]. CRC rates have increased by 5% in the last decade, which is attributed to lifestyle, diet, increased detection via bowel cancer screening programmes [2] and higher incidence of cancer in young individuals (25–49 years old) [1]. Sadly, a quarter (26%) of patients are diagnosed with metastatic disease, with 5-year overall survival at 8% [2]. Of patients treated with curative intent, 50% will develop recurrent disease within 2 years. The increasing cancer burden and escalating costs of diagnosing and managing cancer are not sustainable for public health structures, particularly considering the expense of new targeted treatments and immunotherapies. Greater emphasis on preventive strategies, including the use of therapies to reduce the number of cancer cases [3, 4] is eagerly sought.

Approximately 10% of adenomas develop into CRC [5]. The progression from adenoma to CRC is a multistep process through the accumulation of oncogenes and tumour suppressor gene mutations which can take from years to decades to occur [6, 7]. This is an ideal time to consider preventive strategies, which may stop or reverse the development of CRC. Risk factors for adenoma progression to cancer include large adenoma size, villous type histology, and increased levels of dysplasia [8]. DNA mismatch repair deficiency (dMMR) is observed in 12% of sporadic CRCs [9], 3% of inherited cancers [9], and 10% of adenomas [10]. These tumours have a higher mutational load than microsatellite proficient lesions leading to a robust immune response [11] which can be potentiated by immunotherapy agents such as Nivolumab or Pembrolizumab [12]. Immunotherapy alone or in combination with chemotherapy has transformed the way cancers such as melanoma, renal cell cancer, and non-small cell lung cancer are treated [13]. DNA damage from chemotherapeutic drugs such as cisplatin via adduct formation and paclitaxel via impairing spindle formation increases mutational load resulting in increased cell death. Tumour neoantigens are formed which are considered to be the consequence of genetic alterations accumulating in cancer cells; these antigens result in a more robust immune response than without chemotherapy. However this approach is limited to treatment of established cancers due to toxicity [14, 15]. Utilizing the immune system to prevent cancers is an emerging area of research. Dietary agents such as curcumin [16] and resveratrol [17] or repurposed drugs such as aspirin [18] and metformin [19] have been reported to target the immune system and have the added benefit of eliciting far fewer adverse reactions than many cancer therapies.

Assessing the influence of drug interventions on the immune system in preclinical models remains challenging. Commonly used models have limitations which impact on translation to a clinical setting [20]. Patient-derived organoids rely on cellular disassociation, which destroys the tumour microenvironment [21]. Patient-derived xenografts are undertaken in immunocompromised mice [22], and replacement of the human stroma with mouse stroma is often observed which may influence drug activity [23]. Humanized mouse xenograft systems offer intact immune systems but are expensive, slow to develop and have engraftment limitations [24].

To overcome these limitations and complement existing preclinical assays, we have optimized a workflow for obtaining and culturing patient tissues, termed patient-derived explants (PDEs). Patient relevant drug responses and mechanisms of action can be evaluated using multiple phenotypic, functional, and pharmacodynamic biomarkers, and endpoint analyses whilst retaining the original tumour architecture, microenvironment, and intrinsic immune components [25]. *Ex vivo* models have not been widely adopted. This may be due to the difficulty of obtaining patient tissues, which requires close working between surgery, pathology, research laboratories with coordinated infrastructure, and appropriate ethical and quality assurance standards in place. We have previously shown how culture of CRC explants can aid the examination of mechanisms of action of the dietary agent resveratrol [26], and our work with non-small cell lung cancer has demonstrated the clinical predictive nature of the platform [27].

Here, we present a pilot study assessing the use of multiplexed immunofluorescence (mIF) in combination with colorectal tissue PDEs to evaluate curcumin in a cancer prevention setting. Curcumin is a bright yellow polyphenol compound found in turmeric, obtained from the rhizome of *Curcuma longa*, commonly used as a culinary spice. Despite low systemic bioavailability, an oral dose coats the surface epithelium, generating high local concentrations in colonic tissue, which renders it a good candidate for the management of CRC. A number of randomized controlled trials tentatively support curcumin use in cardiovascular disease, diabetes, and cancer prevention [28]. However, insufficient clinical evidence coupled with a lack of recognized surrogate endpoint biomarkers prevents widespread adoption. Potential biomarkers of the immunomodulatory effects of curcumin, for which there is mounting preclinical data include T helper cells, T regulatory cells, interleukin 8, and high sensitivity CRP [29–31].

In this study, we report preliminary insights into the direct effects of curcumin on the immune response using mIF with PDEs. Immune response has been measured using CD3 as a marker for T cells and CD20 as a marker for B cells. Additionally, immune cell density and localization are assessed. This is an emerging area which may give insight into how immune cells respond to therapy within the tumour or stroma.

## Materials and methods

### Recruitment and consent

Fresh adenoma and CRC tissue were obtained following surgical colorectal resection from six consented patients with no prior chemotherapy or radiotherapy exposure (Research Ethics Committee number: 14/WA/1166). For adenoma tissue, one sample was taken from a patient who had a resection for a large adenoma, the second sample was obtained from a patient who had a CRC and an adenoma in a secondary site within the colon.

An outline of sample and patient characteristics is provided in Table 1. Although the present study is too small to examine associations between measured response and many of these characteristics, they are included here as an example of the data set that should be collected in larger studies of this type. Age, sex, and site of disease are listed as parameters allowing correlation with clinical or immune response. Information on the Tumour Node Metastasis (TNM) classification allows standard grading of tumour infiltration, progression to lymph nodes and any metastatic spread, with a higher stage being associated with more advanced disease and poorer outcomes [32]. This is important as there is emerging data to support a change in increasingly hostile immune microenvironment as CRCs advance [33]. Mutational profile influences the choice of therapy and may influence immune response; patients with rat sarcoma (RAS) wild-type tumours can be offered targeted epidermal growth factor receptor (EGFR) therapies in the metastatic setting, improving patient outcomes [34], whilst BRAF mutant tumours can now be treated with BRAF inhibitors in combination with EGFR targeted therapies also [34]. Both these mutations are assessed in the metastatic setting routinely in the English National Health Service (NHS). MMR status may also be carried out prior to adjuvant chemotherapy or in the metastatic setting if considering immunotherapy treatments [34]. These tests would not routinely be carried out in adenoma tissues in an NHS setting. Lastly, CRC has been classified into four distinct consensus molecular subtypes (CMSs) which are clinically significant in terms of patient survival. They were identified by transcriptomic and gene expression profiling of thousands of patient samples. CMS 1 is characterized by tumours that are BRAF positive and dMMR tumours, CMS 2 are canonical tumours with high levels of WNT and MYC activation and poorly immunogenic, CMS 3 often harbour metabolic dysregulation e.g. lipogenesis and KRAS mutations, and CMS 4 are mesenchymal tumours which are inflamed [35, 36].

**Table 1.** Characteristics of patients and colorectal tissue used for PDEs. – mismatch repair,

Tissue type	Age	Sex	Side of cancer	TNM	BRAF/RAS status	MMR status	CMS subtype
Cancer	80	F	Right	T3N0	NA	MSI	NA
Cancer	89	M	Right	T3N1	NA	MSI	NA
Cancer	64	F	Right	T3N2M1	BRAF—WT/RAS—WT	NA	2/3
Cancer	45	F	Right	T1N1	NA	NA	4
Adenoma	64	F	Left	NA	NA	NA	NA
Adenoma	68	M	Left	NA	NA	NA	4

Overview of patient samples used. BRAF, v-raf murine sarcoma viral oncogene homolog B; CMS, consensus molecular subtype; F, female; M, male; MMR, mismatch repair; MSI, microsatellite instability; NA, no result available; RAS, rat sarcoma; TNM, tumour, node, metastasis; WT, wild type.

### *Ex vivo* explant culture

Adenoma and CRC tissue were placed into Hank’s balanced salt solution (Gibco, UK) and cut into 2–3 mm^3^ pieces [colorectal adenoma patient-derived explants (CRA PDEs) or colorectal cancer patient-derived explants (CRC PDEs)] using two skin graft blades on a dental wax surface. Tissue pieces were then randomly allocated to different wells, with a total of 6–9 pieces of tissue/PDEs per well. The PDEs were placed onto a polyvinylidene fluoride culture insert disc (Millipore, UK) and floated on 1.5-ml fresh culture medium (DMEM with 2% FCS and 1% Glutamax) and incubated at the air liquid interface for 24 h at 37°C and 5% CO_2_ in a humidified atmosphere to recover. CRC or CRA PDEs were then transferred on the filter disc to fresh media and treated with a range of curcumin (Sigma, UK) concentrations (0–10 µM) or vehicle alone as the control, for a further 24 h. Curcumin was initially dissolved in dimethyl sulfoxide at a concentration of 0–10 mM, so that the final concentration of organic solvent in the incubations was 0.001%. A separate well was used for each curcumin concentration. CRA and CRC PDEs were fixed in 10% (w/v) paraformaldehyde for 20 h, then transferred to sponges soaked in 70% (v/v) ethanol and placed in histology cassettes prior to embedding in paraffin blocks from which 4 µM sections were cut. Ideally a piece of tissue should also be obtained at the time of surgery and processed immediately to provide a baseline formalin-fixed paraffin-embedded (FFPE) sample that can be used to assess background levels of cell viability and identify any issues associated with the culture methods or tissue integrity. However, due to limited sample size and sharing of tissue between different projects this was not possible in the present study.

### Histological analysis and mIF

Haematoxylin and eosin (H&E) staining of FFPE sections was conducted using standard approaches by Core Biotechnology Services, University of Leicester. mIF was performed as previously described in detail [37, 38] and antibodies were paired with Opal fluorophores (Akoya Biosciences, UK) as follows: CD20Cy (DAKO, Clone L26, 1:200) (Opal 620); CD3 (DAKO, A0452, 1:200) (Opal 520); cPARP (Abcam [E51] 1:2000) (Opal 480); Ki67 (DAKO MIB1, 1:1000) (Opal 570); Cytokeratin (DAKO, Clone AE1/AE3, 1:400) (Opal 690). Whole slides were scanned using a Vectra Polaris (Akoya Biosciences, UK) in MOTIF imaging mode using a 20× objective lens. Images were then analysed using Inform (V2.4), where tissue was segmented into tumour, stroma, necrotic areas, and background/glass. Subsequently, individual stains were assessed, and a merged view obtained (all stains together with DAPI). Phenotyping for Ki67^+ve^ (proliferation), cPARP^+ve^ (apoptosis), CD3^+ve^ (T cell), CD20^+ve^ (B cell), and DAPI^+ve^ (negative) cells was carried out. All six to nine pieces of tissue were treated as a whole for each well/concentration i.e. additive results. A mean was then derived from the six patient samples to provide a combined result.

### Statistics and phenoptr

R for Windows 4.0.0 with packages phenoptr, tidyverse, ggpubr, and clinfun and GraphPad Prism 9 were used to visualize data. For statistical analysis, Kruskal–Wallis, supplemented with a Mann–Whitney *U* test was performed and *P* < .05 was considered significant. For correlation analysis, Pearson was performed, with *P* < .05 being significant. Trend tests were performed using the Jonckheere–Terpstra trend test, *P* < .05 was significant. For mean values (e.g. proliferation and apoptosis), the mean of each explant derived from a single patient for each drug treatment was calculated, to take into account intra-patient variability in drug response i.e. the mean of 6–9 pieces of tissue/PDEs per well.

## Results

### Workflow of mIF staining for CRA and CRC PDE analysis

To assess CRA and CRC PDE viability and immune cell composition, mIF staining was performed on FFPE sections for protein markers of proliferation (Ki67^+ve^), apoptosis (cPARP^+ve^), T cells (CD3^+ve^), and B cells (CD20^+ve^) with cytokeratin as a tumour marker and DAPI^+ve^ to identify cell nuclei. Many additional antibodies can be used to refine T cell and B cell subsets giving greater granularity to the immune response characterized. However, this can limit assessment of additional features concurrently in the same tissue section such as levels of apoptosis, proliferation, and cytokeratin staining. For this initial proof of concept pilot, additional markers for subsets of T cells e.g. T regulatory cells or cytotoxic T cells were not used.

**Figure 1.**
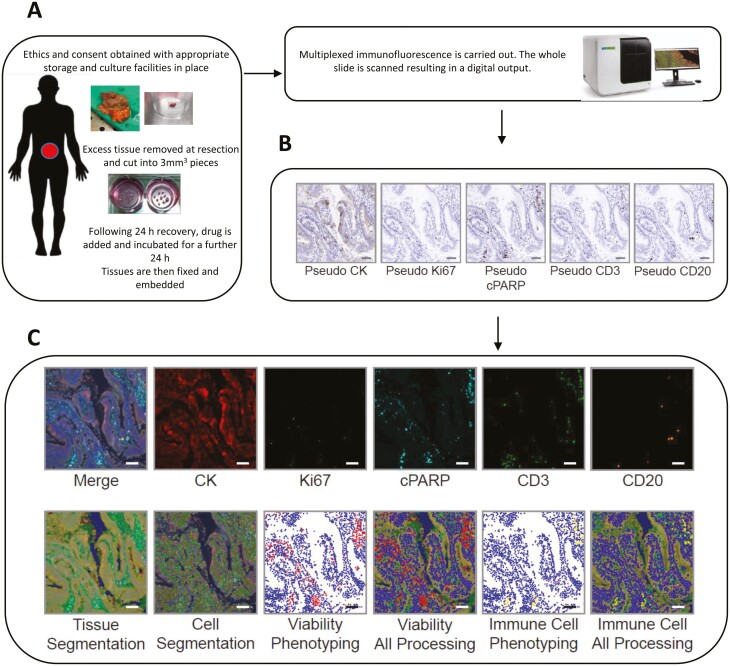
Workflow diagram demonstrating method for evaluation of proliferation, apoptosis, necrosis, and immune cell composition in CRA and CRC PDEs. (A) Excess adenoma or CRC tissue is obtained at surgical resection and cultured as described (B) InForm generated pseudo-DAB images from individual fluorescent markers. (C) Merged (all markers) and individual fluorescent makers are shown at 20× magnification demonstrating cytokeratin [40], Ki67 (yellow), cPARP (cyan), CD3 (green), and CD20 (orange) (top row). Tissue segmentation was carried out in InForm: tumour (yellow), stroma (green), necrosis [40] and background/glass (blue), followed by cell segmentation (for colour figure refer to online version). Viability phenotyping and immune cell phenotyping were performed in InForm separately (bottom row)..

Slide scanning and digital analysis were performed as previously reported [37, 38]. Briefly, following resection, a piece of adenoma or CRC tissue was removed by a pathologist ensuring patient care was not compromised. This sample was then cultured, processed to produce FFPE sections, stained, and scanned (Fig. 1A). InForm software uses individual fluorescence signals to visualize images that appear like 3,3-diaminobenzidine (DAB) stained slides; these can be seen as ‘pseudo-DAB’. Representative examples of each antibody are shown (Fig. 1B). Pseudo-DAB images allow the staining to be observed in a traditional manner. A representative image of all six merged markers is shown (Fig. 1C). InForm built-in machine learning algorithms were trained to segment the tissue into areas of tumour (yellow: CK^+ve^/DAPI^+ve^), stroma (green: CK^−ve^/DAPI^+ve^), necrosis (red: cPARP^+ve^/DAPI^−ve^), and background/glass slide (blue) (Fig. 1C, tissue segmentation image). Next, individual cells were identified and segmented based on DAPI^+ve^ staining (Fig. 1C, cell segmentation image). PDE viability was determined by phenotyping into Ki67^+ve^, cPARP^+ve^, and DAPI^+ve^ only (Fig. 1C, viability phenotyping). Afterwards, this phenotyping was layered with tissue and cell segmentation layers (Fig. 1C, viability all). Immune cell phenotyping was performed by phenotyping on CD3^+ve^, CD20^+ve^, and DAPI^+ve^ only cells (Fig. 1C). Subsequently, this was layered with tissue and cell segmentation layers (Fig. 1C, immune cell all). Analysis was carried out by two authors to ensure data fed into Inform was accurate (S.K. and C.D.).

### Curcumin induces tumour apoptosis in CRA and CRC PDEs

**Figure 2.**
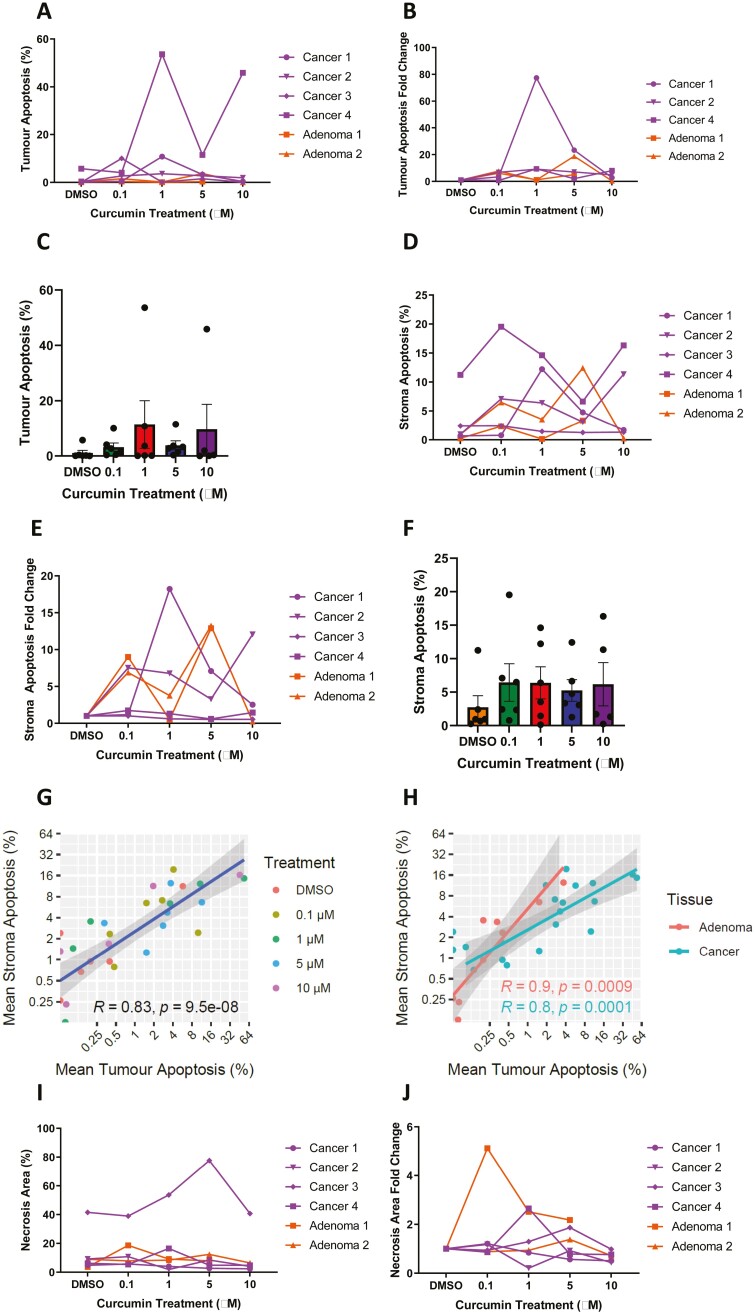
Curcumin induces tumour and stromal apoptosis in CRA and CRC PDEs. Adenoma and CRC tissues were cultured with various concentrations of curcumin (0–10 µM) for 24 h prior to being processed for FFPE. mIF was performed. Curcumin induced higher levels of tumour apoptosis in the six patient samples treated (>2.5-fold change in at least two different curcumin concentrations compared with solvent control) (A–C). Curcumin induced higher levels of stroma apoptosis in four PDEs treated (>2.5-fold change in at least two different curcumin concentrations compared with solvent control) (D–F). A significant relationship between tumour apoptosis and stromal apoptosis regardless of curcumin concentration in CRA and CRC PDEs is observed (*P* < .001). This relationship was also independent of tissue type (G and H). One CRC PDE had high levels of necrosis defined as PARP^+ve^/DAPI^−ve^ areas (I). One CRA PDE was sensitive to curcumin-induced necrosis (>2.5-fold change compared with solvent control in at least two different curcumin concentrations). There was no change observed in other PDEs (J). Each point represents the mean value for all PDEs derived from a single patient sample i.e. the mean of 6–9 pieces of tissue/PDEs per well. Correlation analysis was carried out using Pearson’s correlation, *P* < .05 was significant.

Six patient samples were used in this exploratory study; four CRC samples and two CRA samples. Details on the patient demographics and tissues obtained are presented in Table 1. Tissues were cubed and cultured as described in the Methods. After 24 h recovery, curcumin was added at a range of clinically achievable concentrations (0–10 µM). Classification of tissues was based on the following: tumour CK^+ve^/DAPI^+ve^, stroma CK^−ve^/DAPI^+ve^, and necrosis cPARP^+ve^/DAPI^−ve^. Samples were assessed for viability by quantifying levels of tumour and stroma apoptosis (cPARP^+ve^). Background levels of apoptosis detected in CRC tissues that were processed and fixed immediately after surgical resection have previously been reported to be between 0.2%–8.2% and 0%–6%, which is consistent with the range found in vehicle control samples analysed here (0%–5.7%) [39, 40] (Fig. 2A). This observation suggests tissues retained their viability during 48 h of incubation, as the degree of apoptosis with culture was in keeping with published literature. Ideally, viability should be correlated with uncultured individual patient samples.

Following CRA and CRC PDE culture with curcumin, an increase in tumour apoptosis of >2.5-fold compared with solvent control was seen in at least two different curcumin concentrations in all six samples. A >2.5-fold increase is in keeping with previous work [27]. There was heterogeneity in terms of the level of apoptosis induced as well as the concentration of curcumin inducing highest levels of apoptosis, reflecting heterogeneity across tumours. Notably, cancer patients 1 and 2 were sensitive to curcumin-induced tumour apoptosis at all concentrations tested (Fig. 2A–C). When considering stroma apoptosis, a greater heterogeneity of response was observed. Following curcumin treatment, tissues from cancer patients 1 and 2 and adenoma patients 1 and 2 exhibited an increase in stroma apoptosis of >2.5-fold compared with solvent control with at least two different curcumin concentrations. However, no change was observed in cancer patients 3 and 4. Only cancer patient 2 was sensitive to curcumin-induced stroma apoptosis at all concentrations tested (Fig. 2D–F). Nevertheless, this is the first demonstration that curcumin is able to induce cell death responses in stromal areas of intact human tissues in at least some samples. Interestingly, adenoma tissue was sensitive to both curcumin-induced tumour and stromal apoptosis. An increase in levels of apoptosis following curcumin treatment is consistent with our previous findings in which PDEs derived from CRC liver metastases were treated with curcumin and samples from five out of eight patients showed increased levels of apoptosis relative to their respective control [41].

There was a linear relationship between tumour and stromal apoptosis regardless of curcumin concentration in CRA and CRC tissues (*P* < .001). This relationship was also independent of tumour or stroma tissue type in cancer or adenoma tissue (Fig. 2G and H) suggesting that curcumin-induced apoptosis is indiscriminate in the cell types targeted. Necrosis was evaluated in PDEs using the PARP^+ve^/DAPI^−ve^ population. Evaluation of necrosis demonstrated one cancer sample had higher levels of necrosis compared with other PDEs. Change in the PARP^+ve^/DAPI^−ve^ population following curcumin treatment, revealed only the adenoma from patient 1 was sensitive to curcumin-induced necrosis as defined by >2.5-fold change compared with solvent control in at least two different curcumin concentrations. For all other patient samples little change was observed (Fig. 2I and J).

### Low levels of proliferation are observed in CRA and CRC PDEs

**Figure 3.**
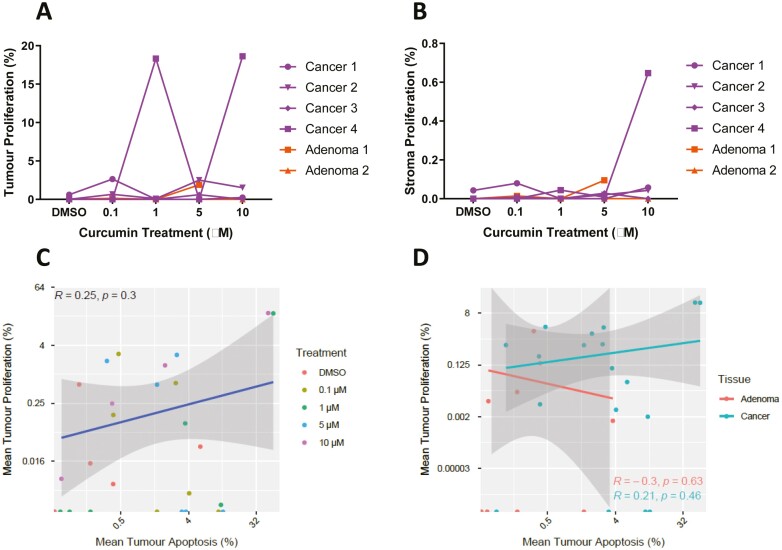
Low levels of proliferation are observed in CRA and CRC PDEs. Adenoma and CRC tissues were cultured with various concentrations of curcumin (0–10 µM) for 24 h prior to being processed for FFPE. Minimal change in levels of tumour proliferation was observed following curcumin treatment of CRA and CRC (A). Low levels of stromal proliferation were detected (<0.65%) (B). No significant relationship between proliferation and apoptosis was observed regardless of curcumin concentration or tissue type (C and D). Each point represents the mean value for all PDEs derived from a single patient sample i.e. the mean of 6–9 pieces of tissue/PDEs per well. Correlation analysis was carried out using Pearson’s correlation, *P* < .05 was significant.

To evaluate the effects of curcumin on proliferation in CRA and CRC PDEs, Ki67 levels were derived as detailed in the methods section. Levels of tumour proliferation were very low in control conditions (<2.6% in all cases except two areas of tissue where 18% was observed). There was no significant difference in Ki67 expression between vehicle control and curcumin-treated CRA or CRC PDEs. There was minimal stromal proliferation detected in any sample (<0.65%) (Fig. 3A and B). There was no relationship between tumour apoptosis and tumour proliferation (Fig. 3C and D).

### Solvent-treated control levels of B cell (CD20^+ve^) and T cell (CD3^+ve^) density correlate with increased curcumin-induced tumour apoptosis in CRA and CRC PDEs

Infiltrating B cells (defined as CD20^+ve^ cells) in lower stage CRC tumours are significantly associated with an improved disease-free survival [42]. Additionally, a positive correlation between B cells and CD8^+ve^ T cells has been reported, with authors suggesting a cooperative-prognostic effect [42]. We therefore sought to use mIF to identify CD20^+ve^ (B cells) and CD3^+ve^ (T cells), to investigate the effect of curcumin on immune cell density and localization, and to correlate this with c-PARP as a biomarker of response.

**Figure 4.**
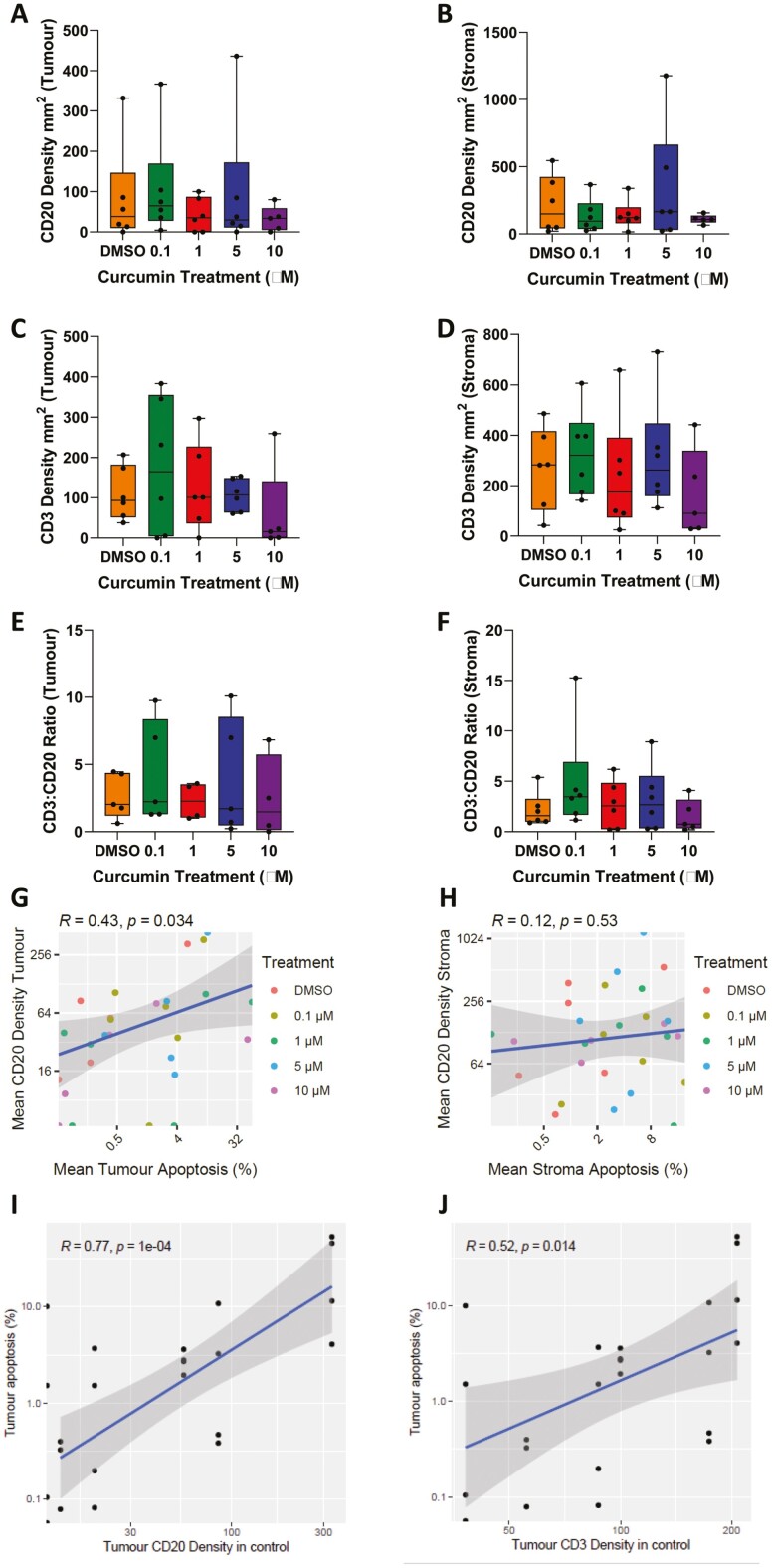
Immune cell composition in CRC and CRA PDEs correlates with curcumin-induced apoptosis. Adenoma and CRC tissues were cultured with various concentrations of curcumin (0–10 µM). Following *ex vivo* culture, density/mm^2^ of CD20^+ve^ and CD3^+ve^ cells were calculated. No change in CD20 or CD3 density or the ratio of CD3:CD20 cells was observed following curcumin treatment in the tumour (A–C) or stroma (D–F). Each point represents the mean density/mm^2^ of each PDE, the central line of the boxplot represents the median of the distribution and the top and bottom represent the first and third quartiles, respectively. The error bars represent the range of values. There was a significant relationship between tumour CD20 density (in curcumin-treated PDEs) and tumour apoptosis following curcumin treatment (*P* < .05) (G), with no relationship between stroma CD20 density and stroma apoptosis (H). Solvent control CD20 and CD3 tumour density correlated significantly with curcumin-induced tumour apoptosis (*P* < .001) (I) and (*P* < .05) (J). Each point represents the mean value for all PDEs derived from a single patient sample i.e. the mean of 6–9 pieces of tissue/PDEs per well. Correlation analysis was carried out using Pearson’s correlation, *P* < .05 was significant.

Following *ex vivo* culture, the density of CD20^+ve^ and CD3^+ve^ cells was calculated for each incubation in terms of the number of cells/mm^2^. Solvent-treated control samples were used to assess the impact of curcumin, however it would have also been useful to compare to uncultured samples taken directly from the surgical specimens to assess the impact of culture and solvent on immune cell viability, activity, and movement. Following curcumin treatment, there was no difference in immune cell density compared with solvent-treated control in the tumour (Fig. 4A and B) or stroma (Fig. 4D and E). This suggests that, at clinically achievable concentrations, immune cells persist. Notably, CD3^+ve^ T cells were more abundant than CD20^+ve^ B cells in CRA and CRC PDEs in the tumour and stroma, as illustrated by the CD3:CD20 ratio which was >1 for all solvent control samples except for cancer patient 4 (Fig. 4C and F). This result is similar to the profile of these cell types previously observed in CRC primary and liver metastasis tissues obtained at surgical resection or diagnostic biopsy [42, 43]. There was no change in CD3:CD20 ratio following curcumin treatment (Fig. 4C and F). However, there was a significant relationship between tumour CD20 density and tumour apoptosis across all samples regardless of curcumin treatment (*P* < .05) (Fig. 4G). In contrast, there was no relationship between stroma CD20 density and stroma apoptosis (Fig. 4H) or CD3^+ve^ T cells and CD3:CD20 ratio and tumour cell death (data not shown) across all samples regardless of curcumin treatment.

An important aspect of an *ex vivo* platform such as PDEs is the ability to predict patient response. We correlated solvent control levels of CD20 and CD3 with tumour apoptosis after curcumin treatment as a biomarker of response. We found there was a significant correlation between CD20 and CD3 density in the tumour regions in control samples and curcumin-treated samples (*P* < .001 and *P* < .05, respectively) (Fig. 4I and J). This shows that higher baseline CD20 and CD3 density is associated with higher curcumin-induced tumour cell death, with the greatest response to curcumin observed in the more immunogenic tumours.

### Curcumin treatment significantly decreases the distance between T cells (CD3^+ve^) and cytokeratin^+ve^ cells

**Figure 5.**
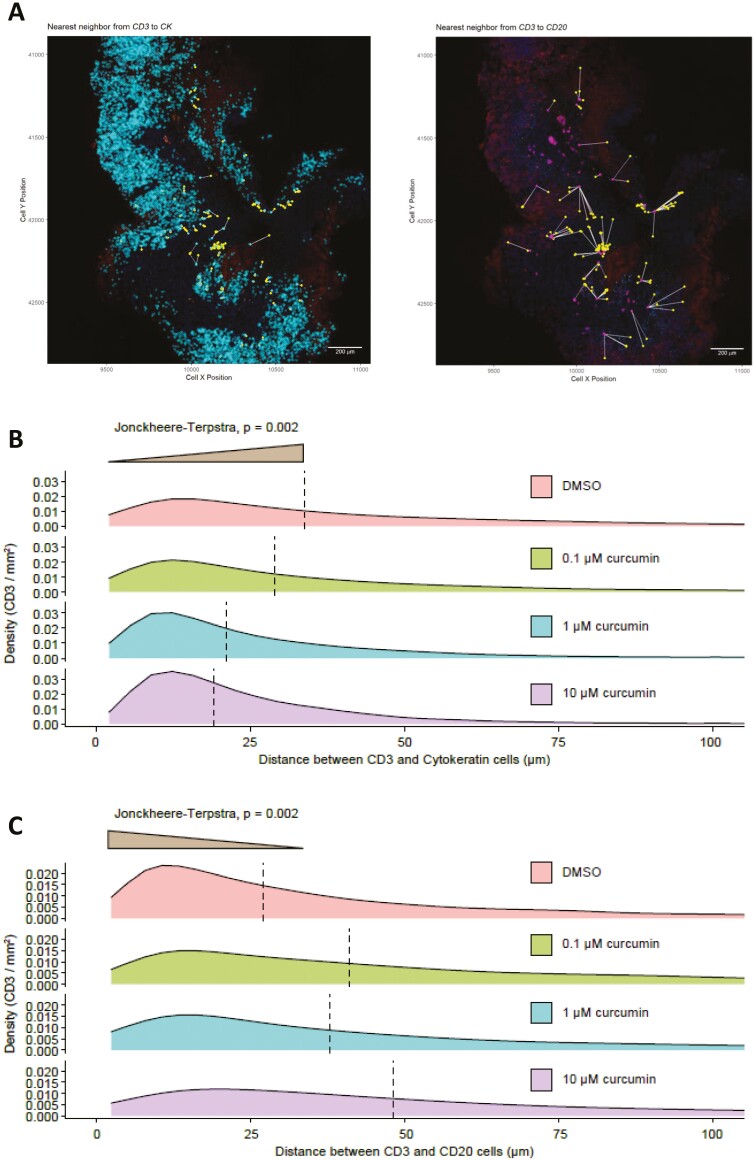
Curcumin changes immune cell localization in CRA and CRC PDEs. Adenoma and CRC tissues were cultured with various concentrations of curcumin (0–10 µM). Following *ex vivo culture*, the density of CD20^+ve^ and CD3^+ve^ cells was calculated. (A) Nearest neighbour analysis from CD3^+ve^ to cytokeratin^+ve^ and CD3^+ve^ to CD20^+ve^ was calculated and representative plots taken from a CRC PDE are shown. (B) Curcumin induced a concentration-dependent significant trend of CD3^+ve^ cells moving towards cytokeratin^+ve^ tumour cells (*P* < .002) when comparing the distribution of distances across treatment groups. The dashed line represents the median. (C) Curcumin induced a concentration-dependent significant trend in increasing the distance between CD3^+ve^ and CD20^+ve^ cells (*P* < .002) when comparing the distribution of distances across treatment groups. The dashed line represents the median.

Using mIF and digital analysis, it is possible to calculate the distance between specific cell populations (Fig. 5A). When paired with PDEs, immune cell migration in response to drug treatment can be evaluated [25]. Curcumin induced a significant concentration-dependent migration of CD3^+ve^ T cells towards cytokeratin^+ve^ tumour cells (*P* < .002) i.e. T cells within a PDE migrated towards tumour cells (Fig. 5B). As epithelial cells stain for cytokeratin, which are on the surface of the lining of the colon, this suggests movement towards the invasive margin of a tumour rather than central tumour. This has been associated with an improved prognosis in patients with CRC [44].

### Curcumin treatment significantly increases the distance between T cells (CD3^+ve^) and B cells (CD20^+ve^)

The role of tumour infiltrating B cells (CD20^+ve^) is controversial. A cooperative effect has been reported between CD20^+ve^ B cells and CD8^+ve^ T cells which is linked to an improved prognosis in CRC, i.e. when cells are close together [45]. However, CD20^+ve^ B cells have also been linked with suppressing antitumour activity, encouraging tumour growth and polarizing macrophages towards an immunosuppressive phenotype [43]. Consequently, the distance between CD3^+ve^ T cells and CD20^+ve^ B cells was assessed. Curcumin induced a concentration-dependent significant increase in distance between CD3^+ve^ T cells and CD20^+ve^ B cells (*P* < .002) (Fig. 5C).

## Discussion

The immune system is critical to the outcome of patients with CRC [46]. An immunosuppressive environment develops during the sequence between transformation of healthy mucosa to adenoma and subsequent CRC [33]. Preventive agents or cancer treatments which can manipulate this may be of patient benefit. For instance, immunotherapies such as Nivolumab or Pembrozulimab have demonstrated an improved overall survival in patients with dMMR CRCs [47]. Due to toxicities, this approach is not generally suitable in a cancer prevention setting.

Utilizing *ex vivo* methods to help understand the role of the immune system in CRC initiation and development could provide insights on how to harness its use for patient benefit. This is key to identifying new immunotherapy targets as well as building upon existing therapies. Here, we have used curcumin, a candidate for CRC therapeutic prevention to pilot the development and utility of CRA and CRC PDE cultures. A total of six samples were used in this pilot study; more samples would allow greater depth and breadth of analysis, however patient samples particularly adenomas, are challenging to obtain as tissues resected are often small (<1 g). Nevertheless, our results suggest that even this low sample number may enable power calculations allowing appropriate sample size for hypothesis testing in the future, depending on marker and magnitude of change expected. Additionally, as FPPE blocks are obtained following therapeutic intervention, hypothesis testing can be carried out *post hoc*. It is noted that cancer prevention agents are likely to be taken by people for prolonged periods, therefore, ideally our culture system should allow repeated dosing over longer durations to mimic chronic exposure [20]. However, this is not currently possible and requires advances in culture methods that allow longer term maintenance of PDE integrity. It is worth recognizing however that, for some biomarkers such as measurements of apoptosis and proliferation, demonstration of an effect after just a short *in vitro* exposure may be indicative of preventive efficacy *in vivo* with longer term interventions [20, 26]. An additional limitation of our study is the lack of a piece of tissue that was processed immediately to provide a baseline FFPE sample, allowing assessment of the impact of culturing and solvents on immune cell viability, activity, and movement. This has been carried out in a similar study using breast cancer PDEs (*n* = 6) in our Centre where no difference in CD4^+ve^/CD8^+ve^ or T regulatory cell was observed between cultured and uncultured tissues (Miles *et al*., unpublished work).

The effect of curcumin on PDEs was assessed using concentrations known to be achievable within the human colorectum [48]. This is key to ensuring outcomes are applicable to a clinical setting. Curcumin increased levels of apoptosis in the tumour of six out of six patient samples and in the stroma of four out of the six samples. This is the first time that stromal apoptotic responses to curcumin have been documented in intact human tissues. Adenoma tissues were sensitive to curcumin-induced tumour and stroma apoptosis, which may be particularly relevant in a cancer prevention setting. The tumour:stroma ratio is a strong independent prognostic tool in epithelial cancers such as colon cancer, with higher levels of stroma being associated with a poorer prognosis [49] and chemotherapy resistance [50]. In the present study a linear response was not observed for individual patients, in that the maximal increases in apoptosis were apparent at the lower concentration range, whilst higher concentrations often failed to elicit a >2.5-fold change relative to solvent control. This finding may further challenge the preconception that ‘more is better’ when selecting doses of dietary-derived compounds for use in cancer prevention studies [26] and highlights the difficulties around choosing an optimal dose for therapy. Due to the lack of clear dose–response relationship, it is important to consider penetration of curcumin through a PDE. Data from our group have previously shown for CRC liver metastasis PDEs that no significant differences were observed in measures of proliferation (Ki67) and apoptosis (caspase-3) when comparing sections closest to the media/bottom of tissue with sections closer to the air interface/top of tissue. Ideally, this type of analysis should be replicated with each PDE, however limitations of tissue may prevent this [51].

Following culture, CRA and CRC PDEs demonstrated a denser population/mm^2^ of T cells (CD3^+ve^) compared with B cells (CD20^+ve^) in solvent control PDEs i.e. the CD3:CD20 ratio was >1, which is consistent with published literature in CRC and CRC liver metastasis [45, 43]. In addition, the density of CD20 and CD3 in solvent control PDEs correlated significantly with curcumin-induced tumour apoptosis. This may indicate some tumours are intrinsically more likely to be sensitive to curcumin-induced apoptosis based on initial levels of CD20 and CD3, which has not been previously reported. It is unclear if tumour or patient characteristics also impact on the likely sensitivity of a tissue to curcumin-induced apoptosis, but this would be of interest. More broadly, some tumours are considered hot or cold to immune-based therapies i.e. sensitive or resistant, and there is keen interest in how a cold tumour could be induced to become hot [52]. This correlation was not carried out on tissue prior to PDE culture, so it requires further investigation and verification to ascertain whether the CD3:CD20 ratio in tissues processed immediately after biopsy or resection can be used to predict response to curcumin without the need for *ex vivo* culture.

Curcumin significantly decreased the distance between CD3^+ve^ T cells and cytokeratin^+ve^ cells, suggesting migration towards the invasive margin of the tumour. Patients with a higher T cell burden in the invasive margin compared with the central tumour have a higher disease-free survival compared with those with lower infiltration in the primary CRC tumour [44]. A similar pattern is observed in CRC liver metastasis. In liver metastasis a barrier is created around T cells at the epithelium by tumour associated macrophages (defined as CD163^+ve^) which express programmed cell death ligand 1 (PD-L1) and a reduction in cytotoxic T cells. This immunosuppressive hurdle may need to be overcome by successful immunotherapies [43]. It is possible that curcumin is able to modulate this hurdle, although it would be important in future studies to compare the effects of curcumin with uncultured tissue samples to ensure the changes we observed are not a consequence of culturing alone.

Curcumin significantly increased the distance between CD3^+ve^ T cells and CD20^+ve^ B cells in a concentration-dependent manner. The role of B cells is controversial. Higher expression is associated with a favourable prognosis, although absolute numbers in CRC are low compared with those of CD3^+ve^ T cells [43, 45]. Conversely, B cells have been associated with suppressing antitumour activity and polarizing macrophages towards an immunosuppressive phenotype. Therefore, the observed modulation by curcumin of CD3^+ve^ T cells away from CD20^+ve^ B cells may encourage a more favourable immune milieu for an antitumour response. The movement of cells seen here with curcumin (~10–20 µm) was not as great as that observed with Nivolumab (~90 µm) [25], however the toxicity profile is more favourable.

Future work should involve recruitment of further patients to expand the sample set and consideration of different immune cell subsets e.g. adaptive (CD8^+^ T lymphocytes, FOXP3^+^ T regulatory cells) as well as the innate immune system (CD68^+^ macrophages and CD66b^+^ neutrophils). Further molecular subtyping may help to identify those most likely to respond to therapy versus those unlikely to respond. Homogenization of PDEs would allow more detailed analysis of protein, DNA, RNA, and/or metabolite profiles using a range of targeted and ‘omic techniques. Such analyses may both provide insights into mechanisms of action and identify patients likely to benefit from therapy.

In summary, the *ex vivo* model described here could help discover effective therapies for cancer prevention, elucidate modes of action, and identify predictive biomarkers of response that can be used to stratify individuals to specific therapies in a precision prevention approach.
